# Detrimental effect of maternal and post-weaning high-fat diet on the reproductive function in the adult female offspring rat: roles of insulin-like growth factor 2 and the ovarian circadian clock

**DOI:** 10.1007/s10815-017-0915-5

**Published:** 2017-04-17

**Authors:** Yu-Ju Lin, Ching-Chou Tsai, Li-Tung Huang, Jiunn-Ming Sheen, Mao-Meng Tiao, Hong-Ren Yu, Chih-Cheng Chen, You-Lin Tain

**Affiliations:** 1 Department of Obstetrics and Gynecology, Kaohsiung Chang Gung Memorial Hospital and Chang Gung University, College of Medicine, Kaohsiung, Taiwan; 2 Department of Pediatrics, Kaohsiung Chang Gung Memorial Hospital and Chang Gung University, College of Medicine, 123 Dabi Road, Niausung, Kaohsiung, 833 Taiwan; 3 Institute for Translational Research in Biomedicine, Kaohsiung Chang Gung Memorial Hospital and Chang Gung University, College of Medicine, Kaohsiung, Taiwan

**Keywords:** High-fat diet, Steroidogenesis, Insulin-like growth factor 2, Circadian rhythm

## Abstract

**Purpose:**

We evaluate the impact of maternal and post-weaning high-fat (HF) diet on ovarian follicular population, steroidogenesis, and gene expression with a focus on the circadian clock system and insulin-like growth factor 2 (*Igf2*) in adult offspring ovaries, and to elucidate whether a maternal and post-weaning diet confers similar risks.

**Methods:**

Virgin Sprague-Dawley rats were fed with normal chow (C) diet or HF diet for 5 weeks before mating, during gestation, and lactation. Female offspring were fed with the C or HF diet from weaning to 6 months of age, resulting in four study groups (*n* = 6 per group): C/C, C/HF, HF/C, and HF/HF.

**Results:**

Ovaries from offspring exposed to post-weaning HF diet (i.e., the C/HF and HF/HF groups) had a decrease in small follicle numbers, but with similar numbers of antral follicles and corpora lutea. Offspring from HF-fed dams (i.e., the HF/C and HF/HF groups) had increased plasma estradiol concentrations and decreased luteinizing hormone levels at 6 months of age. In addition, *Igf2* and each of the circadian rhythm core genes *Clock*, *Per1*, *Per2*, and *Per3* were increased in the ovaries of offspring exposed to maternal HF diet (both HF/C and HF/HF groups).

**Conclusions:**

Maternal and post-weaning HF diet programs the reproductive profile of the female offspring in adult life through different manners. Post-weaning HF intake resulted in the reduction of small follicles in adulthood, whereas maternal HF diet had long-term deleterious consequences on female offspring steroidogenesis and coincided with alteration of the upregulation of the imprinted gene *Igf2* and changes in ovarian circadian rhythms.

**Electronic supplementary material:**

The online version of this article (doi:10.1007/s10815-017-0915-5) contains supplementary material, which is available to authorized users.

## Introduction

Widespread consumption of high-energy foods has increased dramatically over the last century and is linked to a global burden of metabolic syndrome and associated comorbidities, including reproductive dysfunction and infertility [1, 2]. In addition, there is increasing evidence that nutrition in pregnancy and early life play an essential role in placental development, fetal growth, and organogenesis, and it can elicit long-term effects on the health of offspring, through a process known as developmental programming [3, 4]. Our previous research has shown that a postnatal high-fat (HF) diet aggravated prenatal stress to induce programmed hypertension and liver steatosis [5–7]. Additionally, previous studies showed that diet-induced maternal obesity or a post-weaning HF diet can cause early onset of puberty and estrous cycle abnormalities in female progeny [8–10]. However, limited data are available on the long-term effects of HF exposure during gestation, lactation, and early life, especially on ovarian follicular development and steroidal hormones [11–13].

Insulin-like growth factor 2 (*Igf2*), which is one of the best known epigenetically imprinted genes, and a key intra-ovarian regulator of follicular development and steroidogenesis [14, 15], is associated with greater body weight, obesity [16, 17], and polycystic ovary syndrome [18, 19]. Previous studies had shown that parental obesity influence *Igf2* by epigenetic changes which could alter the metabolic health of the fetus [20, 21]; however, how the locally intra-ovarian *Igf2* is programmed by maternal and post-weaning HF intake is unknown. The circadian timekeeping system is actively engaged in the maintenance of normal metabolism and reproductive function [22, 23]. In mammals, a central pacemaker located in the suprachiasmatic nuclei of the anterior hypothalamus coordinates the timing of these rhythms. The molecular basis of circadian oscillations is composed of positive and negative transcriptional–translational feedback loops [22, 24]. Dimers of the CLOCK and BMAL1 proteins bind to a specific E-box promoter to induce the transcription of core clock genes *Period* (*Per1*, *Per2*, and *Per3*) and *Cryptochrome* (*Cry1* and *Cry2*), which produce proteins that form dimers after translation to repress their own transcription by competing with CLOCK/BMAL1 binding. Following degradation of the inhibitory proteins, the transcription–translation loop starts over for another circadian cycle. The contribution of central circadian clock gene to the reproductive physiology and steroidogenesis is well known [22, 23]. Diets high in fat or sugar have been shown to alter circadian clock function [25]. Circadian clocks are also present in the cells of the ovary [11, 26, 27], uterus [28], and placenta [29]. Nevertheless, the role of peripheral oscillators in the ovary or uterus remains unclear. It has been proposed that a multi-oscillatory circadian system with synchronization between central and peripheral components is necessary for proper timing of female reproduction function [30, 31]. Disruption of this synchrony might contribute to the onset or progression of various reproductive pathologies [30].

Taken together, the aim of this study was to evaluate the impact of maternal and post-weaning HF consumption on ovarian follicular development and steroidogenesis in adult female offspring. Additionally, we intended to elucidate whether a post-weaning or maternal HF diet confers similar risks and the interaction between two factors on the reproductive function in female progeny, with a focus on the circadian clock system and *Igf2* in the offspring ovaries.

## Materials and methods

### Animals and experimental design

Our animal protocol was approved by the Institutional Animal Care and Use Committee of the Chang Gung Memorial Hospital (Approval Number 2015031105). Virgin Sprague-Dawley (SD) rats (*n* = 12, 7 weeks old) were purchased from BioLASCO Taiwan Co., Ltd. All animals were housed in an animal facility at 22 °C, with a relative humidity of 55%, in a 12 h light/12 h dark cycle, with food and sterile tap water available ad libitum.

Female rats were weight-matched and assigned to receive either a normal rat chow diet (27.5% protein, 59.7% carbohydrate, 12.6% fat by energy, 3.25 kcal/g; Fwusow Taiwan Co., Ltd., Taichung, Taiwan) or HF diet (#D12331; 16.4% protein, 25.5% carbohydrate, 58% fat by energy, 5.24 kcal/g; Research Diets, New Brunswick, NJ, USA) for 5 weeks before mating and during gestation and lactation. Body weights were recorded weekly. At 14 weeks of age, the female rats were allowed to mate with male SD rats that were fed a normal chow diet for 3 days. After mating, pregnancy dams (control *n* = 3, HF *n* = 2) were allowed to deliver their pups, and litter size was standardized to 11 pups (with equal numbers of each sex, whenever possible) to standardize the quantity of milk and maternal care received by each offspring. The day of birth was designated postnatal day 0 (PND 0). After delivery and lactation period, the female rat offspring were weaned at PND 21 onto either the normal chow (C) or HF diet from weaning to 6 months of age, resulting in four experimental groups (maternal diet/post-weaning diet): C/C, C/HF, HF/C, HF/HF (*n* = 6 for each group). The number of animals in each group were calculated using the resource equation method based on the law of diminishing return, following the procedure described by Charan and Kantharia [32], in which *E* (degree of freedom of ANOVA) is calculated as *E* = total number of animals − total number of groups. In this study, *E* = 24 − 4 = 20. At PND 180, the female offspring were sacrificed at 4 h into the light period to assess various fat deposits (including retroperitoneal, mesenteric, and subcutaneous fat pad) and ovary weights. Heparinized blood samples were collected. One ovary was fixed in 10% formalin in neutral-buffered solution and processed for histological analysis, while the other was immediately frozen in liquid nitrogen and stored at −80 °C for further analysis.

### Histological analysis of the ovaries

Immediately after the rats were sacrificed, the ovaries were harvested, stored in saline on ice, and dissected from the surrounding tissues. Then, the ovary was fixed in 10% formalin in neutral-buffered solution, pH 7.4 (Wako Junyaku, Osaka, Japan). Four-micrometer-thick sections were made and stained with hematoxylin and eosin (H&E) for morphometric analysis of structure and follicle distribution. Images were captured using a digital camera (CTR 5000; Leica, Wetzlar, Germany) and a Nikon Eclipse E600 microscope (×10 magnification).

The slice was examined in duplicate in a blinded fashion by two examiners (Y.C.-L. and Y.J.-L.) and only follicles containing an oocyte with a visible nucleus were counted. We focus on the developing follicular population, which is crucial to maintain the reproductive function. Therefore, follicles showing evidence of atresia were excluded. A follicle was considered to be undergoing atresia when pyknotic granulosa cells were observed or whenever the oocytes showed obvious signs of degeneration (e.g., deformed shape, vacuolation, loss of nuclear membrane, and/or fragmentation). Follicles were classified according to the following characteristics [33]: primary follicles had an oocyte surrounded by one layer of cubical granulosa cells; secondary follicles had two or more layers of granulosa cells with no antral space; antral follicles had an oocyte surrounded by several layer of granulosa cells with one or more independent antral spaces, or with a cumulus granulosa cell layer; and finally, cystic follicles were devoid of oocytes and displayed a large antral cavity, a well-defined thecal cell layer, and a thin (mostly monolayer) granulose cell compartment containing apparently healthy cells. We categorized primary and secondary follicles as small follicles because they were gonadotropin-independent in this very early preantral phase [34, 35]. The total volume of each section was calculated (area × thickness of the section), and the follicle counts for each sample were normalized by the total volume (μm^3^) and expressed per 10 mm^2^.

### Plasma hormones

Plasma luteinizing hormone (LH) and follicle-stimulating hormone (FSH) were analyzed using a MILLIPLEX MAP Rat Pituitary Magnetic Bead Panel (Millipore, Billerica, MA, USA). The intra-assay coefficients of variations for the LH and FSH assays, respectively, were 3.3 and 2.8%. Plasma estradiol (E_2_) was analyzed using a MILLIPLEX MAP Steroid/Thyroid Hormone Magnetic Bead Panel (Millipore, Billerica, MA, USA) and the intra-assay coefficients of variation were less than 10%.

### Quantitative real-time PCR analysis

RNA was extracted using a previously described procedure [36]. Two-step quantitative real-time PCR (qPCR) was conducted using Quantitect SYBR Green PCR Reagents (Qiagen, Valencia, CA) on an iCycler iQ Multi-color Real-Time PCR Detection System (Bio-Rad, Hercules, CA). Several components of circadian clock genes analyzed in this study included *Clock*, *Bmal1*, *Period* genes (*Per1*, *Per2*, and *Per3*), *Cryptochrome* genes (*Cry1* and *Cry2*), and *Rev-erbα*. The imprinted genes *Igf2* and insulin-like growth factor 2 receptor (*Igf2R*) were also determined. Ribosomal 18S was used as a reference in all analyses. Primers were designed using GeneTool Software (Biotools, Edmonton, Alberta, Canada) (Table S1). All samples were run in duplicate. For the relative quantification of gene expression, the comparative threshold cycle (*C*_T_) method was employed. The averaged *C*_T_ was subtracted from the corresponding averaged r18S value for each sample, resulting in Δ*C*_T_. ΔΔ*C*_T_ was achieved by subtracting the average control Δ*C*_T_ value from the average experimental Δ*C*_T_. The fold change was established by calculating 2^−ΔΔCT^ for experimental versus reference samples.

### Statistics

Normally distributed data are presented as the mean ± S.E.M. Data from the C/C, C/HF, HF/C, and HF/HF offspring groups were compared using one-way analysis of variance (ANOVA) with LSD post hoc test for multiple comparisons. A two-way ANOVA analysis was also conducted to assess the main effects of the diets and the interaction between maternal diet and post-weaning offspring diet. A *P* <0.05 was considered statistically significant. All analyses were performed using the Statistical Package for the Social Sciences software (SPSS; IBM, Armonk, NY, USA).

## Results

### Adult offspring bodyweight, fat mass, ovary weight, and liver weight

After 5 weeks of exposure to the diets before mating, the mean body weight of dams was not significantly different between the two groups (262.6 ± 8 g vs. 283.5 ± 7 g for control and HF diet group, respectively; *p* = 0.200). After delivery, litter sizes were not significantly affected by maternal diet exposure (pups per litter—control = 10.3 ± 2.6; HF = 12.5 ± 1.5). The mortality rate in female pups was not different among the four groups. We observed that female pup body weight, abdominal circumference, and ovary weight were not different among the groups at 6 months old (Table 1). There was an effect of the post-weaning HF diet on the total body fat mass (including retroperitoneal, mesenteric, and subcutaneous fat pad) per 100 g body weight (Table 1, two-way ANOVA, *p* = 0.048), where a 1.3-fold increase in fat mass was observed in the C/HF group compared with the C/C group. There was no significant effect of the maternal diet or interaction between the maternal and post-weaning HF diet on the fat mass. The liver weight was markedly heaviest in the HF/HF group (*p* = 0.027, one-way ANOVA post hoc LSD analysis), and there was an effect of the offspring diet on the liver weight (two-way ANOVA, *p* = 0.007), which showed that post-weaning HF diet resulted in an increased liver weight in the offspring. However, there was no effect of the maternal diet or an interaction between the maternal diet and post-weaning HF diet.

**Table 1:** Body weight, ovary, and fat mass in offspring rats from four groups

Group	C/C	C/HF	HF/C	HF/HF	Two-way ANOVA/*F*(1,20)		
					*P*_Mat_	*P*_Offs_	*P*_Int_
Body weight (g)	362 ± 15	367 ± 20	355 ± 16	382 ± 11	0.815	0.349	0.504
AC (mm)	186 ± 4.7	189 ± 5.4	187 ± 4.3	198 ± 3.4	0.275	0.134	0.438
Ovary weight (mg)	73.3 ± 8	88.3 ± 7	73.3 ± 4	86.7 ± 7	0.905	0.053	0.905
Fat mass/100 g BW	6.8 ± 0.8	8.9 ± 0.5	7.6 ± 0.8	8.4 ± 0.6	0.840	0.048*	0.363
Liver weight (g)	10.5 ± 0.9^a^	12.3 ± 1.2	9.5 ± 0.5^b^	14.5 ± 1.6^ab^	0.599	0.007**	0.180

Values are the means ± S.E.M. *n* = 6/group. Figures sharing the same superscript letter (a, b) are statistically significantly different at *p* < 0.05 by one-way ANOVA with LSD analysis post hoc

*C/C* offspring from maternal C diet with post-weaning C diet, *C/HF* offspring from maternal C diet and fed post-weaning HF diet, *HF/C* offspring from maternal HF diet and fed post-weaning C diet, *HF/HF* offspring fed from maternal and post-weaning HF diet, *AC* abdominal circumference

*p < 0.05, **p < 0.01, comparing effects of maternal diet (*P*_Mat_), offspring diet (*P*_Offs_), and interaction (*P*_Int_) by two-way ANOVA

### Plasma hormone profile

**Fig. 1:**
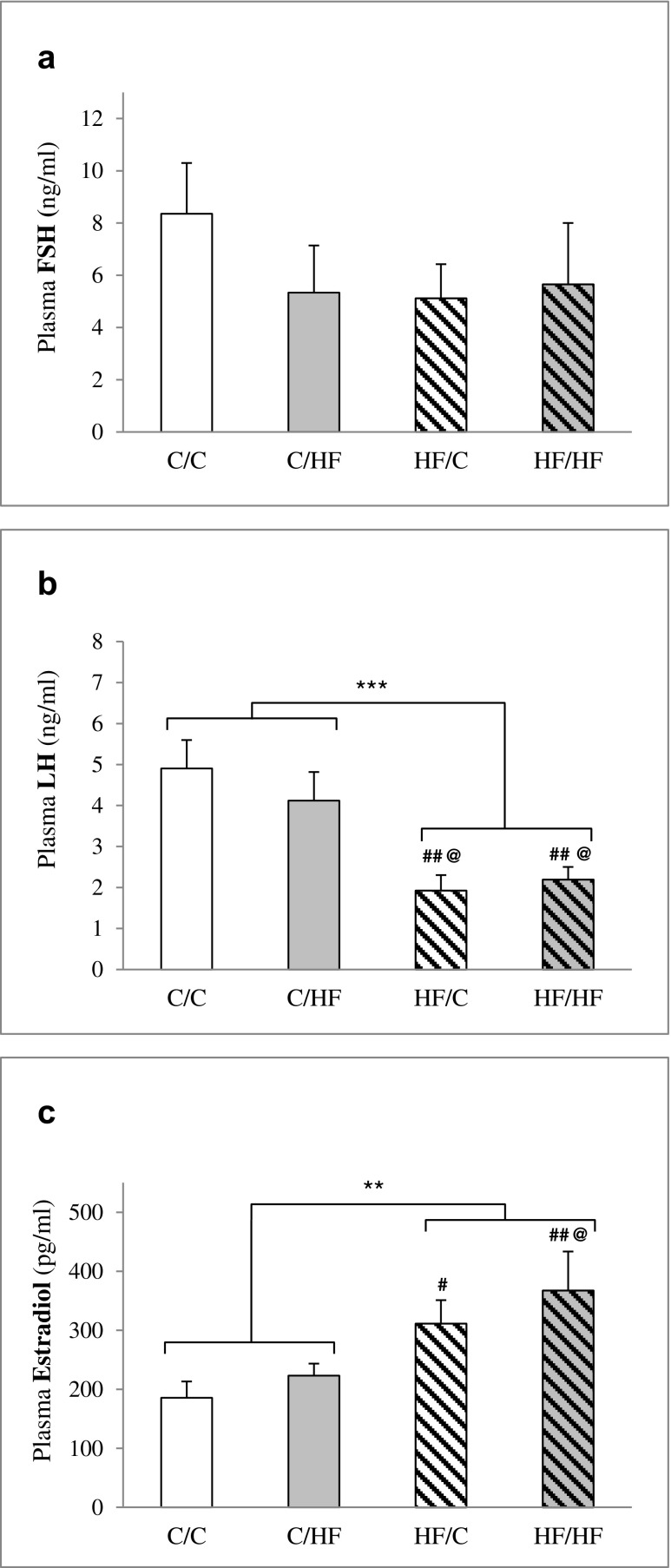
Effects of maternal and/or post-weaning HF diet on plasma hormone profile in female offspring rats at 6 months old. Values are the means ± S.E.M. *p < 0.05, **p < 0.01, ***p < 0.001 by comparing effects of maternal diet, offspring diet, and interaction (two-way ANOVA); #*p* < 0.05, ##*p* < 0.01 compared to C/C; @*p* < 0.05 compared to C/HF by one-way ANOVA with LSD analysis post hoc, *n* = 6/group

We found little measureable effect on the plasma FSH levels among the four groups (Fig. 1a). There was an effect of the maternal diet on plasma LH (two-way ANOVA, *p* < 0.001, Fig. 1b), such that offspring from HF-fed dams (i.e., the HF/C and HF/HF groups) showed a significant decrease in plasma LH. There was no effect of the offspring diet or an interaction between the maternal diet and post-weaning HF diet on plasma LH. The maternal HF diet caused a significant increase in plasma E_2_ (two-way ANOVA, *p* = 0.004, Fig. 1c); moreover, maternal and post-weaning HF synergistically caused a highest estradiol level (with a 1.9-fold increase in HF/HF vs. C/C, *p* = 0.006, one-way ANOVA post hoc LSD analysis). There were no effects of post-weaning HF diet on the plasma E_2_ levels.

### Gene expression studies

**Fig. 2:**
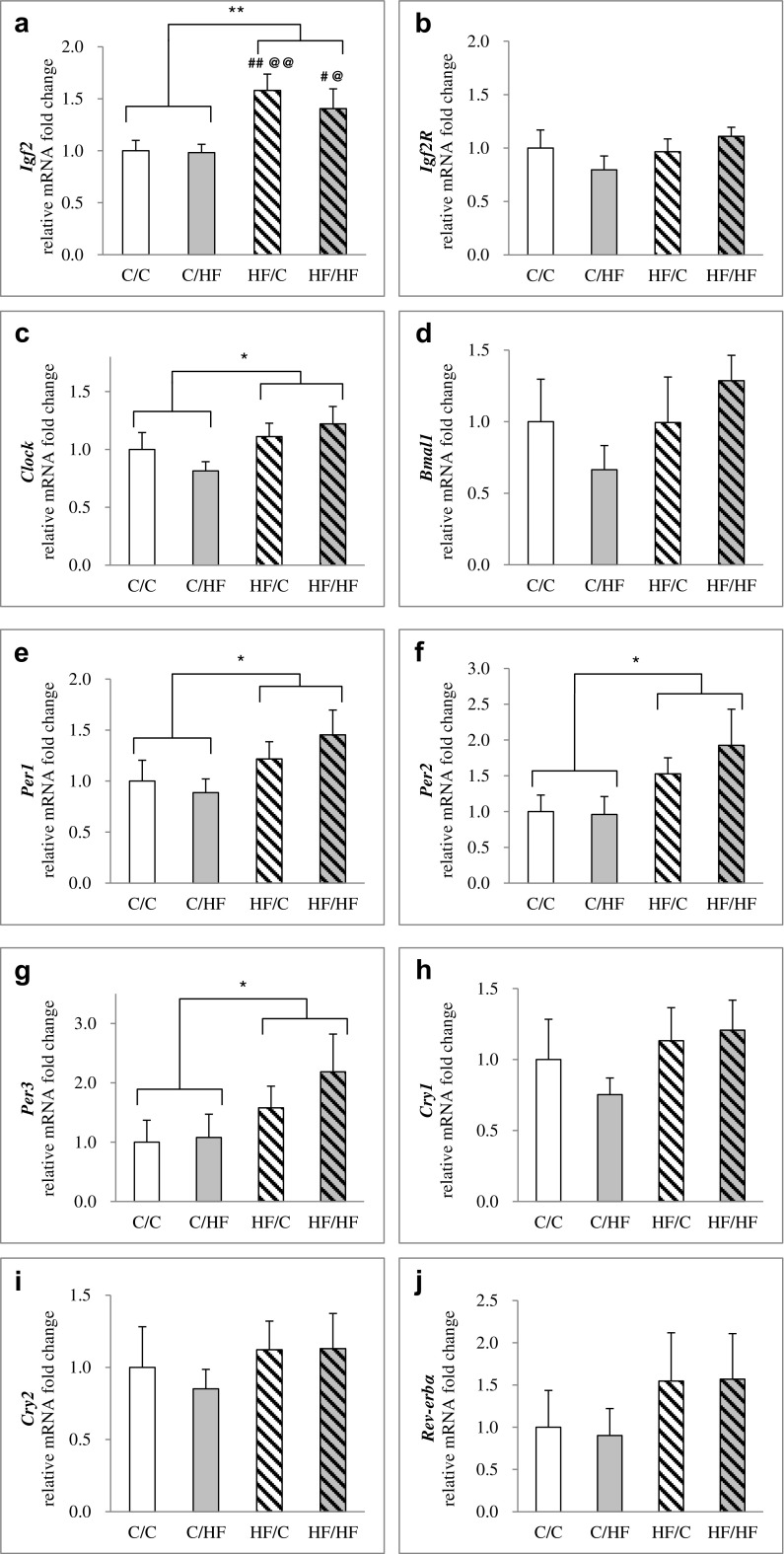
Effects of exposure to maternal and/or post-weaning HF diet on gene expression of imprinted genes *Igf2* and the circadian clock genes in the female offspring ovaries at 6 months old. **a** *Igf2*, **b** *Igf2R*, **c** *Clock*, **d** *Bmal1*, **e** *Per1*, **f** *Per2*, **g** *Per3*, **h** *Cry1*, **i** *Cry2*, and **j** *Rev-erbα.* Values are expressed as relative mRNA fold changes normalized to the C/C group by calculating 2^−ΔΔCT^. All values are the means ± S.E.M. *p < 0.05, **p < 0.01 by comparing effects of maternal diet, offspring diet, and interaction (two-way ANOVA), #*p* < 0.05, ##*p* < 0.01 compared to C/C; @*p* < 0.05, @@*p* < 0.01 compared to C/HF by one-way ANOVA with LSD analysis post hoc, *n* = 6/group

The mRNA expression of the imprinted genes *Igf2*, *Igf2R*, and the circadian clock genes in the ovaries is shown in Fig. 2. There was an effect of the maternal diet on *Igf2* expression (two-way ANOVA, *p* = 0.001, Fig. 2a), and that the ovaries in offspring from HF-fed dams (i.e., the HF/C and HF/HF groups) showed a significant increase in expression levels compared with the levels found in the ovaries of offspring from C-fed mothers (i.e., with a 1.58-fold increase in HF/C vs. C/C, *p* = 0.004, one-way ANOVA). There was no effect of the offspring diet or an interaction between the maternal diet and offspring diet on *Igf2* expression in the ovaries. Maternal HF diet was also the main effect for elevated gene expression of the *Clock* genes (two-way ANOVA, *p* < 0.05, Fig. 2c) in the offspring ovaries. In addition, there was also a significant effect of the maternal HF diet on *Per1*, *Per2*, and *Per3* gene expression (Fig. 2e–g). Thus, significant increases in *Per1*, *Per2*, and *Per3* gene expression were found in the ovaries of offspring from HF-fed dams compared with the levels found in the ovaries of offspring from C-fed mothers. There was no effect of the offspring diet or an interaction between the maternal diet and offspring diet on *Per1*, *Per2*, and *Per3* expression in the ovaries. We observed that there were no differences in *Igf2R*, *Bmal1*, *Cry1*, *Cry2*, or *Rev-erbα* mRNA levels among four groups (Fig. 2b, d, h, i, and j).

### Morphometric analysis of ovarian follicles

**Fig. 3:**
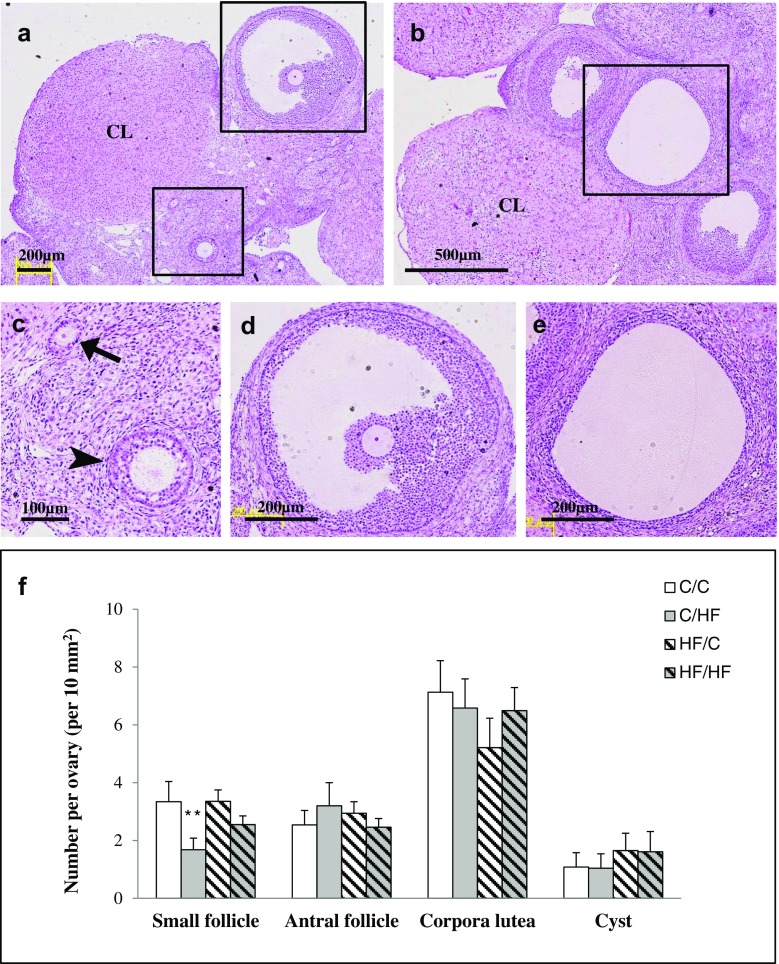
Representative hematoxylin-and-eosin-stained histological image of follicles at different development stages. Ovaries were from rats of 6 months of age. (**a**) Shows the primary follicle (**c**, *arrow*), secondary follicle (**c**, *arrowhead*), and antral follicle (**d**) with corpora lutea; (**b**) shows the cystic follicle (**e**) with corpora lutea. *CL* corpora lutea. (**f**) Effects of maternal and/or post-weaning HF diet on morphometric analysis of ovarian follicles in rats at 6 months old. All values are the means ± S.E.M. Small follicles included primary and secondary follicles. **p < 0.01 by comparing effects of maternal diet, offspring diet, and interaction (two-way ANOVA), *n* = 6/group

We assessed the effect of maternal and post-weaning HF diet on ovarian follicle morphologic differentiation by histology at PND 180. Representative images of H&E-stained follicles at each stage are shown in Fig. 3. Our results showed that the four groups had comparable numbers of antral follicles, cystic follicles, and corpora lutea at 6 months of age. However, there was an effect of the offspring diet on the number of small follicles (including primary and secondary follicles) in the ovaries of the offspring (two-way ANOVA, *p* < 0.01, Fig. 3f); a 2.0-fold reduction was observed in the C/HF group compared with the C/C group. We observed that maternal HF diet had no effect on the number of small follicles.

## Discussion

Our study provides insight into a novel mechanism by which maternal and post-weaning HF diet programs the reproductive potential of the female offspring in adult life. The major findings are as follows: (1) post-weaning HF consumption contributes more fat mass and liver weight deposition; (2) maternal HF diet had a major effect on steroidal hormone changes, which caused a significantly higher plasma E_2_ concentration and lower LH level in the offspring of HF-fed dams (i.e., the HF/C and HF/HF groups), and maternal and post-weaning HF synergistically caused a highest estradiol level in HF/HF group; (3) maternal HF diet had the main effect on gene expression of the imprinted gene *Igf2* and the circadian clock genes, which increased *Igf2*, *Clock*, *Per1*, *Per2*, and *Per3* mRNA expression in offspring ovaries; and (4) post-weaning HF diet did not cause any changes in the numbers of antral follicles, cystic follicles, or corpora lutea but did reduce the number of small follicles.

In the current study, we observed that female offspring body weight was not different among the four groups at 6 months. HF diets are often used to promote obesity in rodents; however, some authors did not find statistically differences in body weight [37]. Nevertheless, we noted that post-weaning HF diet has a major impact on the total body fat mass and liver weight despite the maternal and post-weaning HF elicited little effect on metabolic syndrome-like conditions (e.g., obesity and abdominal circumference) on the female offspring.

Two critical processes in ovarian biology are the assembly of the primordial follicles early in development and the subsequent development and transition of the primordial follicle to the primary follicle. These processes directly affect the number of oocytes available to a female throughout her reproductive life [38]. In the neonatal rodent, the process of follicular assembly is precisely coordinated and takes place at birth, while in humans this occurs during the fetal period [39]. The embryonic rodent ovary contains no follicles, and the oocytes are arranged in large clusters called nests. Immediately after birth, selected oocytes undergo a wave of apoptosis, and surplus surviving oocytes form the primordial follicle pool; the entire process is completed within 4 days of the postnatal period [38]. Kezele et al. developed a hypothesis that high levels of maternal and fetal steroids, including both progesterone and estradiol in the ovary, act to arrest primordial follicle assembly and development and the decline in steroid levels late in fetal development or after birth allows the initiation of primordial follicle assembly and development [40]. This endocrine system has likely evolved to prevent the premature and inappropriate development of primordial follicles. A previous study has shown that maternal obesity increases serum E_2_ at PND1, PND7, and until PND 60 in the female offspring [13]. We also found higher plasma E_2_ levels in 6-month-old progeny from HF-fed dams; the higher estradiol may cause abnormal follicular development. Given that oocytes and follicles are continuously lost to atresia, both maternal HF diet and postnatal HF diet had been reported to alter follicular development and accelerate follicle loss or atresia, involving several possible mechanisms [11, 12, 41, 42]. A previous study had reported that cafeteria diet for 60 days in rat may impair the ovulatory process and induce the presence of follicular cysts [12]; however, in the current maternal/post-weaning HF diet model, the number of cystic follicles seem similar among four groups after long-term diet modification for 6 months. This discrepancy may be possibly explained by the different study design and study period. We found that the offspring diet had a major programming effect on the number of small follicles, which suggests that post-weaning HF diet has a major impact on the early follicle pool, but the gonadotropin-dependent large follicles and corpora lutea seem unchanged.

*Igf2* may play a role in reproductive function and is more abundant in the fluid from large follicles [43]. *Igf2* and gonadotropins synergistically stimulate the expression of receptors for these gonadotropins (FSH and LH) in granulosa cells, which further enhances follicular proliferation, maturation, and production of estradiol and progesterone [15, 43, 44]. *Igf2R* may serve as a type of decoy receptor to modulate *Igf2* action, to inactivate it [14]. In the second part of the study, we found that a maternal HF diet caused a significant increase in plasma E_2_ concentration and a decrease in plasma LH level, which may suggest that intra-uterine HF exposure programmed female offspring steroidogenesis in adult life via an *Igf2*-induced upregulation effect. In our study, despite the number of ovarian antral follicles at PND 180 being similar among the four groups, we observed that maternal HF nutrition led to increased *Igf2* mRNA expression in the progeny’s ovaries and increased plasma E_2_ levels. Taken together, we postulate that the increased estradiol level is involved in *Igf2*-induced upregulation of the FSH receptor, rather than through downregulation of *Igf2r*, and the lower plasma LH level in HF-fed dams (i.e., the HF/C and HF/HF groups) may result from a negative feedback loop by inhibiting the production of gonadotropin in the hypothalamus because of high plasma E_2_ status. In addition, diet-induced maternal obesity and hyperestrogenism were both reported with advanced puberty-onset and estrous cycle disruption in adult female offspring [13, 45]. Our study may provide a possible explanation for maternal HF nutrition resulting in hyperestrogenism via an *Igf2*-induced upregulation effect in the ovary, which subsequently causes further female offspring reproductive dysfunction in adult life.

In our study, we found that the circadian genes *Clock*, *Bmal1*, *Period* genes, *Cryptochrome* genes, and *Rev-erbα* are expressed in the rat ovary, which is consistent with previous studies [11, 26, 27]. Our study showed that maternal HF diet exposure had a major effect in altering the expression levels of circadian-clock genes in rat ovaries, similar to previously reported result [11]. Moreover, we observed that expression levels of *Clock* and *Per1–3* were both elevated at the time of sampling (4 h into light period), suggesting the antiphase expression patterns of positive and negative elements in the female offspring were also disrupted by maternal HF diet. The results were similar to but not exactly the same as previous reports. Cheong et al. reported that circadian genes (*Clock* and *Bmal1*) were elevated in the ovaries of 15-week-old C57BL/6J mice from HF-fed dams, whereas lower expression levels of *Cry1* and *Per1* were observed in offspring exposed to HF nutrition throughout life, which preserved the circadian gene phase-related expression pattern [11]. One of the possibilities suggested by both the present study and that of Cheong et al. is the importance of diet composition. We chose a high-fat, high-sucrose diet for 6 months to induce more fat mass and liver weight deposition in the female offspring. Although the maternal diet exposure period is similar, it is possible that the programming effect on offspring by different diet formulas and study periods will have different impacts. Therefore, we found that maternal HF diet changes the expression levels of ovarian circadian genes; it upregulates the *Clock* gene and dysregulates the negative feedback function of *Period* genes to cause local circadian dysfunction. The design of our study had several potential weakness and limitations. First, we did not assess the estrous cycle phase at tissue harvesting, so the possibility of mixing different phases of estrous cycle may mask the actual effect by the HF diet itself. A previous study had demonstrated that *Per1* and *Per2* mRNA expression in rat ovary displayed a rhythmicity with a period of 24 h regardless of estrous cycle phase, as well as in randomly collected cyclic ovaries from rats during continuous darkness [26]. Moreover, literature review had reported that early follicle growth in rodents is very protracted. The follicle grows from primordial follicles to the early antral stage (0.2 to 0.4 mm in diameter) over a period of greater than 60 days. Then the early antral follicles are subjected to cyclic recruitment, and only 2 to 3 days are needed for them to grow into preovulatory follicles [46]. Therefore, unknown estrous cycle phase seems to have little influence on the number of the small follicles, but the number of the large antral follicles may fluctuate. Taken together, that mixture of different estrous cycle phase may have influenced the strength of our data, but it still does not obscure the significance of our findings. Another limitation is that we did not evaluate other parameters of reproductive function (e.g., puberty-onset and estrous cycle anomalies) in response to maternal and post-weaning HF intake. Finally, ovary is one major component of the hypothalamic–pituitary–gonadal (HPG) axis; therefore, whether disturbed local ovarian circadian clock by HF-induced programming might be the consequence or only a part of changes of HPG axis deserves further evaluation to elucidate the role of these clocks in the HPG axis.

## Conclusions

In conclusion, our data indicate that maternal and post-weaning HF diet programs the reproductive profile of the female offspring in adult life through different manners. Post-weaning HF consumption resulted in the reduction of small follicle numbers in rat ovaries, whereas maternal HF diet had long-term deleterious consequences on female offspring steroidogenesis and coincided with alteration of the upregulation of the imprinted gene *Igf2* and changes in ovarian circadian rhythms.

*C*, Chow; *E*_*2*_, Estradiol; *FSH*, follicle-stimulating hormone; *HF*, high fat; *H&E*, hematoxylin and eosin; *HPG*, hypothalamic–pituitary–gonadal; *Igf2*, insulin-like growth factor 2; *Igf2r*, insulin-like growth factor 2 receptor; *LH*, luteinizing hormone; *PND*, postnatal day; *SD*, Sprague-Dawley.

## Electronic supplementary material

Table S1 (DOCX 16 kb)
