# Supplementary material for: Detrimental effect of maternal and post-weaning high-fat diet on the reproductive function in the adult female offspring rat: roles of insulin-like growth factor 2 and the ovarian circadian clock
Source: J Assist Reprod Genet. 2017 Apr 17;34(6):817–26. doi: 10.1007/s10815-017-0915-5 (PMC5445052; doi:10.1007/s10815-017-0915-5)
Supplement: Supplementary file 1 — (DOCX 16 kb) [file 10815_2017_915_MOESM1_ESM.docx]

**Table S1. Primers sequences used for qPCR analysis**

| **Gene** | **Primer sequences (5' to 3')** | **Accession Number** |
| --- | --- | --- |
| ***Igf2*** | Forward 5'- GACGACTTCCCCAGATACCC-3'  Reverse 5'- CACTGATGGTTGCTGGACAT-3' | **NM_** 031511 |
| ***Igf2r*** | Forward 5'- GAGTGGGCTTCCCAGAATATC-3'  Reverse 5'- CGGCACACGTTGAGGTAGTAT-3' | **NM_** 012756 |
| ***Clock*** | Forward 5'- CCACTGTACAATACGATGGTGATCTC -3'  Reverse 5'- TGCGGCATACTGGATGGAAT -3' | **NM_**021856 |
| ***Bmal1*** | Forward 5'- ATTCCAGGGGGAACCAGA -3'  Reverse 5'- GAAGGTGATGACCCTCTTATCCT -3' | **NM**_024362 |
| ***Per1*** | Forward 5'- GCTTGTGTGGACTGTGGTAGCA -3'  Reverse 5'- GCCCCAATCCATCCAGTTGT -3' | **NM**_001034125 |
| ***Per2*** | Forward 5'- CATCTGCCACCTCAGACTCA -3'  Reverse 5'- CTGGTGTGACTTGTATCACTGCT -3' | **NM**_031678 |
| ***Per3*** | Forward 5'- TGGCCACAGCATCAGTACA -3'  Reverse 5'- TACACTGCTGGCACTGCTTC -3' | **NM**_023978 |
| ***Cry1*** | Forward 5'- ATCGTGCGCATTTCACATAC -3'  Reverse 5'- TCCGCCATTGAGTTCTATGAT -3' | **NM**_198750 |
| ***Cry2*** | Forward 5'- GGGAGCATCAGCAACACAG -3'  Reverse 5'- GCTTCCAGCTTGCGTTTG -3' | **NM**_133405 |
| ***Rev-erbα*** | Forward 5'- CTACTGGCTCCCTCACCCAGGA -3'  Reverse 5'- GACACTCGGCTGCTGTCTTCCA -3' | **NM**_001113422 |
